# Rice Senescence-Induced Receptor-Like Kinase (*OsSRLK*) Is Involved in Phytohormone-Mediated Chlorophyll Degradation

**DOI:** 10.3390/ijms21010260

**Published:** 2019-12-30

**Authors:** Na-Hyun Shin, Do Thi Trang, Woo-Jong Hong, Kiyoon Kang, Jadamba Chuluuntsetseg, Joon-Kwan Moon, Yo-Han Yoo, Ki-Hong Jung, Soo-Cheul Yoo

**Affiliations:** 1 Graduate School of Integrated Bioindustry, Sejong University, Seoul 05006, Korea; 2 Department of Plant Life and Environmental Science, Hankyong National University, 327, Jungangro, Anseong-si, Gyeonggi-do 17579, Korea; 3 Graduate School of Biotechnology and Crop Biotech Institute, Kyung Hee University, Yongin 446-701, Korea; 4 Department of Plant Science, Plant Genomics and Breeding Institute, Research Institute of Agriculture and Life Sciences, Seoul National University, Seoul 151-921, Korea

**Keywords:** rice, receptor-like kinase, RNA sequencing, chlorophyll degradation, leaf senescence, phytohormone

## Abstract

Chlorophyll breakdown is a vital catabolic process of leaf senescence as it allows the recycling of nitrogen and other nutrients. In the present study, we isolated rice senescence-induced receptor-like kinase (*OsSRLK*), whose transcription was upregulated in senescing rice leaves. The detached leaves of *ossrlk* mutant (*ossrlk*) contained more green pigment than those of the wild type (WT) during dark-induced senescence (DIS). HPLC and immunoblot assay revealed that degradation of chlorophyll and photosystem II proteins was repressed in *ossrlk* during DIS. Furthermore, ultrastructural analysis revealed that *ossrlk* leaves maintained the chloroplast structure with intact grana stacks during dark incubation; however, the retained green color and preserved chloroplast structures of *ossrlk* did not enhance the photosynthetic competence during age-dependent senescence in autumn. In *ossrlk*, the panicles per plant was increased and the spikelets per panicle were reduced, resulting in similar grain productivity between WT and *ossrlk*. By transcriptome analysis using RNA sequencing, genes related to phytohormone, senescence, and chlorophyll biogenesis were significantly altered in *ossrlk* compared to those in WT during DIS. Collectively, our findings indicate that *OsSRLK* may degrade chlorophyll by participating in a phytohormone-mediated pathway.

## 1. Introduction

Chlorophyll plays a pivotal role in photosynthesis by absorbing light and transferring the light energy and electrons to other molecules [1]. Two types of chlorophylls exist, namely, chlorophyll *a* (Chl *a*) and chlorophyll *b* (Chl *b*). While Chl *a* serves as a component of all chlorophyll–protein complexes, such as photosystem I (PSI), photosystem II (PSII), and cytochrome *b*_6_*f* complex, Chl *b* is found only in the PSI-associated light-harvesting complex I (LHCI) and PSII-associated LHCII, whose apoproteins are encoded by the *Lhca* and *Lhcb* gene families, respectively [2]. LHCI is located in the stroma lamellae of the thylakoid membrane, whereas LHCII is mainly present in the grana, the stacking region of the thylakoid membranes, and its intermolecular interaction is required for the formation and maintenance of grana stacks [3,4].

Chlorophyll degradation is the visual symptom of leaf senescence, which is accompanied by the breakdown of chlorophyll–protein complexes and thylakoid membranes [5]. This irreversible process occurs via sequential reactions catalyzed by the chlorophyll-degrading enzymes at LHCII [6,7]. Initially, Chl *b* is converted to Chl *a* by Chl *b* reductase, which is encoded by non-yellow coloring 1 (*NYC1*) and *NYC1*-like (*NOL*), and by 7-hydroxymethyl Chl *a* reductase (HCAR) [2,8,9,10]. The removal of magnesium (Mg) from Chl *a* is catalyzed by Mg-dechelatase, encoded by Mendel’s green cotyledon genes (*Non-yellowing*/*Stay-green* (*NYE/SGR*) genes) to produce pheophytin *a* (Phein *a*) [11,12]. Phein *a* is then converted to pheophorbide *a* (Pheide *a*) by pheophytin pheophorbide hydrolase (PPH) [13]. The porphyrin ring of Pheide *a* is cleaved by pheophorbide *a* oxygenase (PAO), producing red Chl catabolite (RCC) and thus losing its green color [14,15]. Furthermore, RCC is catalyzed by red Chl catabolite reductase (RCCR) to produce primary fluorescent Chl catabolite (*p*FCC) [16]. This *p*FCC is then transported into vacuoles and isomerized to nonfluorescent Chl catabolite (NCC) [17].

Leaf senescence generally occurs in an age-dependent manner; however, it can be affected by internal and environmental factors, such as phytohormones, pathogen infection, extreme temperatures, salt, drought, nutrient deficiency, and shading [18,19,20]. Thus, genetic regulators controlling phytohormone biosynthesis and signaling affect the onset and progress of leaf senescence in rice. For instance, rice (*Oryza sativa*) mutants with high levels of abscisic acid (ABA), by overexpressing rice *WRKY5* (*OsWRKY5*), promote leaf yellowing during senescence [21]. Impairment of ABA signaling by mutation of *SPOTTED LEAF3* (*SPL3*)-encoding rice mitogen-activated protein kinase kinase kinase 1 (OsMAPKKK1) delays leaf senescence in rice [22]. Overexpression of rice *DNA-binding one zinc finger 24* (*OsDOF24*) represses jasmonic acid (JA) biosynthesis via the downregulation of *OsAOS1*, resulting in delayed leaf senescence [23]. Previous studies have reported that ethylene is associated with leaf senescence. Mutation of *OsPSL*, encoding a core 2/I branching beta-1,6-*N*-acetylglucosaminyl transferase, leads to increased endogenous ethylene level, thereby promoting leaf senescence in the *psl* mutant [24].

Receptor-like kinase (RLK) is one of the largest transcription factor (TF) families and comprises more than 1131 rice members [25]; it is further classified into 44 subfamilies based on the presence of N-terminal extracellular kinase domains. Of these, leucine-rich repeats (LRRs) constitute the biggest subfamily. RLKs are conserved in diverse plant species and implicated in various biological functions, such as plant development and defense; for example, maize (*Zea mays*) crinkly4 controls leaf development [26], Arabidopsis ERECTA regulates organ shape [27], CLAVATA 1 maintains meristems [28], HAESA is involved in floral organ abscission [29], brassinosteroid insensitive 1 (BRI1) and BRI-associated kinase 1 (BAK1) perceive brassinosteroids [30], and carrot (*Daucus carota*) PSKR controls cell proliferation [31]. In addition to the defined roles of RLKs in plant growth, rice (*Oryza sativa*) Xanthomonas resistance 21 (Xa21) enhances tolerance to *Xanthomonas oryzae* pv. *oryzae* [32], and wheat (*Triticum aestivum*) Lr10 locus receptor kinase (LRK10) mediates resistance to leaf rust disease [33].

In contrast to the biological functions of RLKs in plant development and defense responses, the underlying mechanisms of RLKs in leaf senescence are not clearly elucidated in rice. Herein, we report the characterization of *OsSRLK*, a LRR-type *RLK* gene, in regulating chlorophyll degradation and leaf senescence. Results showed that mutation of *OsSRLK* inhibited the degradation of chlorophyll and LHCII proteins during dark-induced senescence (DIS). Transcriptome analysis using RNA sequencing revealed that expression of numerous genes related to phytohormone biosynthesis and signaling was significantly altered in *ossrlk* mutant compared to wild type (WT) under DIS conditions. Thus, *OsSRLK* presumably regulates leaf senescence via regulatory pathways of chlorophyll degradation and phytohormones.

## 2. Results

### 2.1. OsSRLK Is Upregulated during Leaf Senescence

**Figure 1:**
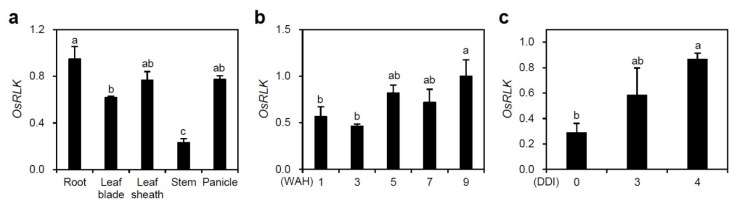
Expression patterns of rice senescence-induced receptor-like kinase (*OsSRLK*). (**a**) *OsSRLK* was differentially expressed in various wild-type (WT) tissues separated from root, leaf blade, leaf sheath, stem, and panicles at the heading stage. (**b**,**c**) Transcript levels of *OsSRLK* were determined in the flag leaves of WT grown in the paddy field under natural long day (NLD) conditions (≥14 h light/day) (**b**) or in the detached leaves of WT grown in the growth chamber for three weeks under long day (LD) conditions (14 h light/10 h dark) (**c**). The transcript levels of *OsSRLK* were determined by RT-qPCR analysis and normalized to those of *OsUBQ5* (AK061988). Mean and standard deviations were obtained from more than three biological replicates. Different letters indicate significant differences according to one-way ANOVA and Duncan’s least significant range test (*p* < 0.05). These experiments were repeated twice and gave similar results. WAH, week(s) after heading; DDI, day(s) of dark incubation.

We initially investigated the spatial expression patterns of *OsSRLK* in the root, leaf blade, leaf sheath, stem, and panicle of the Korean japonica rice cultivar “Donjin” (hereafter termed WT), grown in the paddy field under natural long day (NLD) conditions. *OsSRLK* transcripts showed significantly higher accumulation in root, leaf blade, leaf sheath, and panicle than in the stem (Figure 1a). Moreover, to monitor the change of *OsSRLK* expression during leaf senescence, we measured the transcript levels of *OsSRLK* in attached and detached WT leaves, whose senescence was induced in an age-dependent manner and dark treatment, respectively. The RT-qPCR analysis revealed that *OsSRLK* was significantly expressed in the senescing rice leaves (Figure 1b,c). These results suggest that *OsSRLK* is involved in the onset and progression of leaf senescence in rice. *OsSRLK* comprises 3668 nucleotides, with a 1926 bp open-reading frame encoding a protein made of 641 amino acids. Amino acid sequence alignments between *OsSRLK* and its putative orthologs indicated that the leucine-rich repeat and catalytic domain of serine/threonine kinases were highly conserved among diverse plant species (Figure S1).

### 2.2. ossrlk Mutant Delays Leaf Yellowing during DIS

**Figure 2:**
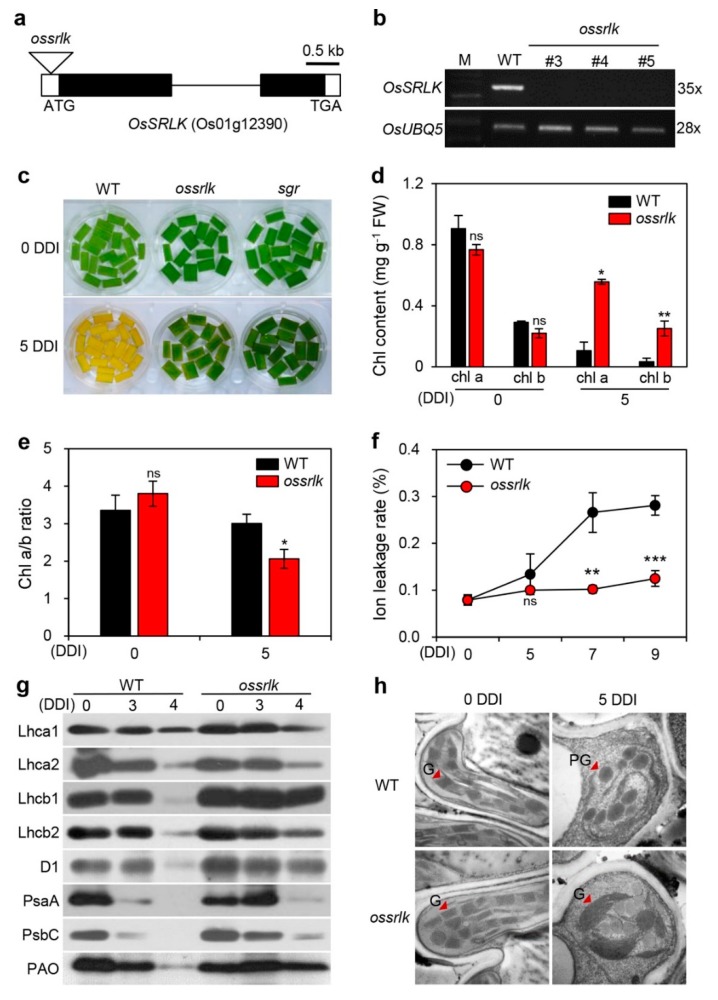
The *ossrlk* mutant shows a delay in leaf yellowing during dark-induced senescence. (**a**) Schematic diagram depicting the positions of T-DNA insertions in *OsSRLK*. Black and white bars represent the exon and untranslated region, respectively. The black line represents the intron. The triangle indicates the *ossrlk* mutant (PFG_1A-15835). (**b**) Mutation of *OsSRLK* verified by semiquantitative RT-PCR. *OsUBQ5* was used as a loading control. The Arabic numerals on the right side of images indicate the numbers of PCR cycles. (**c**–**f**) Detached leaves of WT, *ossrlk*, and *sgr* plants grown in paddy soil for three weeks were incubated in 3 mM 2-(*N*-morpholino)ethanesulfonic acid (MES) buffer (pH 5.8) under complete darkness at 28 °C. (**c**) The leaf yellowing phenotypes were observed at 0 and 5 DDI. Total chlorophyll contents (**d**), Chl *a*/*b* ratios (**e**), and ion leakage rates (**f**) were determined in WT and *ossrlk* during dark incubation. Mean and standard deviation values were obtained from the three biological repeats. Asterisks indicate a significant difference between WT and *ossrlk* mutant (Student’s *t*-test, * *p* < 0.05, ** *p* < 0.01, *** *p* < 0.001). Chl, chlorophyll; FW, fresh weight; ns, not significant. These experiments were repeated thrice with similar results. (**g**) Immunoblotting of detached leaves from WT and *ossrlk* plants at 0, 3, and 4 DDI using antibodies against photosynthetic proteins (Lhca1, Lhca2, Lhcb1, Lhcb2, D1, PsaA, PsbC, and PAO). (**h**) Transmission electron microscopy images were obtained from detached leaves of three-week-old WT and *osdof24-D* at 0 and 5 DDI, as depicted in Figure 2c. These experiments were repeated twice with similar results. G, grana (stack of thylakoids); PG, plastoglobule. Scale bars = 5 μm.

To examine the biological functions of *OsSRLK* in leaf senescence, we obtained a T-DNA insertion line (PFG_1A-15835) from the RiceGE database (http://signal.salk.edu/cgi-bin/RiceGE), in which T-DNA fragment was integrated into the 5′ untranslated region (5′ UTR) of *OsSRLK* (Figure 2a). To verify the expression levels of *OsSRLK* in this mutant line, we identified *OsSRLK* transcripts in the leaves of three-week-old WT and PFG_1A-15835. RT-PCR analysis revealed that, while *OsSRLK* transcripts highly accumulated in WT, they were lost in PFG_1A-15835 due to T-DNA insertion (Figure 2b). These results indicate that PFG_1A-15835 is a knockout mutant (hereafter termed *ossrlk*).

To determine the phenotypic difference between WT and *ossrlk* during dark-induced senescence, we observed the progress of leaf yellowing in the detached leaves of three-week-old WT and *ossrlk* that were incubated in 3 mM 2-(*N*-morpholino)ethanesulfonic acid (MES) buffer (pH 5.8) at 28 °C under complete darkness. The phenotype of *stay-green* (*sgr*), a nonfunctional stay-green mutant, was used for comparison as a positive control. While WT leaves turned yellow at 5 DDI, both *ossrlk* and *sgr* leaves retained their green color (Figure 2c). In accordance with this phenotype, both Chl *a* and Chl *b* were highly accumulated in *ossrlk* compared to WT at 5 DDI (Figure 2d). The ratio of Chl *a* to Chl *b* was significantly less in *ossrlk* compared to that in WT at 5 DDI (Figure 2e). When plant cells are subjected to senescence, their reduced membrane integrity leads to an increase in the ion leakage rate [34]. Thus, ion leakage rate serves as an indicator to evaluate the plant senescence process. As presented in Figure 2f, although the ion leakage rate rapidly increased in WT after 5 DDI, only a slight change was observed in *ossrlk* (Figure 2f). Moreover, we investigated the levels of photosynthetic proteins via immunoblot analysis using antibodies against PSII proteins (antenna: Lhcb1 and Lhcb2; core: D1 and PsbC), PSI proteins (antenna: Lhca1, Lhca2; core: PsaA), and PAO. While PSI proteins were degraded in both WT and *ossrlk* at 4 DDI, the degradation of PSII proteins and PAO were inhibited in *ossrlk* (Figure 2g). Furthermore, we compared the chloroplast structures of *ossrlk* leaf tissues with those of WT at 0 and 5 DDI. Transmission electron microscopy analysis revealed that, at 0 DDI, the chloroplast structure of *ossrlk* was similar to that of WT; however, the thylakoids of *ossrlk* had bigger and thicker grana structures than those of WT at 5 DDI (Figure 2h). These results indicate that null mutation of *OsSRLK* delays leaf senescence by inhibiting the degradation of chlorophyll and PSII-associated LHCII during DIS.

To examine whether *ossrlk* delays leaf yellowing during natural senescence, we observed the senescence phenotypes in WT and *ossrlk* plants grown in the paddy field under natural long day conditions. While no phenotypic differences were observed between WT and *ossrlk* plants at one week after heading (WAH) (Figure S2a), *ossrlk* exhibited delayed leaf senescence phenotype at 8 WAH compared to WT (Figure S2b). We further monitored the leaf greenness and Fv/Fm ratio (efficiency of photosystem II) in WT and *ossrlk* plants grown in the paddy field under natural long day conditions. Based on changes in the SPAD-502 value, which determines leaf greenness, flag leaves of *ossrlk* retained higher green color than those of WT throughout the grain filling period (Figure S2c); however, no significant difference was observed in the change in Fv/Fm between WT and *ossrlk* during natural senescence under field conditions (Figure S2d). These results suggest that *ossrlk* delays leaf yellowing without enhanced photosynthetic capacity during natural senescence.

### 2.3. Overexpression of OsSRLK Accelerates Leaf Yellowing under DIS Conditions

**Figure 3:**
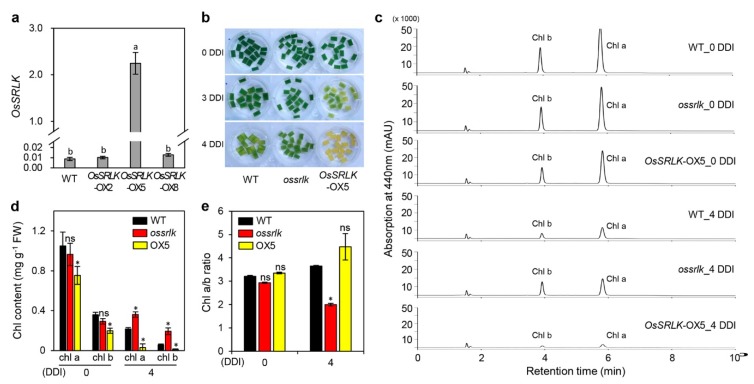
Overexpression of *OsSRLK* promotes chlorophyll degradation during DIS. (**a**) Expression of *OsSRLK* in the flag leaves of WT and transgenic lines grown in the paddy field under NLD conditions. The transcript levels were determined using RT-qPCR and normalized to those of *OsUBQ5*. Mean and standard deviations were obtained from more than three biological replicates. Different letters indicate significant differences according to one-way ANOVA and Duncan’s least significant multiple range test (*p* < 0.05). (**b**–**e**) Detached leaves of WT, *ossrlk*, *OsSRLK*-OX5 plants grown in the paddy soil for three weeks were incubated in 3 mM MES (pH 5.8) under complete darkness at 28 °C. (**b**) The leaf yellowing phenotypes were observed at 0, 3, and 4 DDI. (**c**) HPLC elution profiles of chlorophyll *a* (Chl *a*) and chlorophyll *b* (Chl *b*). Absorbance at 440 nm were measured in detached leaves of WT, *ossrlk*, and *OsSRLK*-OX5 at 0 and 4 DDI. The same weight of detached leaves was extracted with the same volume of 80% acetone. The same volume of extract was loaded in each injection. Measurements of peak areas using HPLC analysis were used for determining the contents (**d**) and ratios (**e**) of Chl *a* and Chl *b*. Mean and standard deviation values were obtained from three biological repeats. Asterisks on *ossrlk* or *OsSRLK*-OX5 indicate a significant difference from WT, as determined by Student’s *t*-test (* *p* < 0.05). ns, not significant; FW, fresh weight. These experiments were repeated thrice with similar results.

To confirm the effects of *OsSRLK* on leaf yellowing, we generated three individual rice transgenic lines harboring *35S::OsSRLK*. RT-qPCR analysis revealed that numerous *OsSRLK* transcripts were expressed in the *OsSRLK*-overexpressed 5 (*OsSRLK*-OX5) transgenic line (Figure 3a). Detached leaves of three-week-old *OsSRLK*-OX5 promoted leaf yellowing at 3 and 4 DDI compared to those of WT (Figure 3b). The HPLC analysis for extracts derived from detached leaves of WT, *ossrlk*, and *OsSRLK*-OX5 plants revealed that, while Chl *a* and Chl *b* was less accumulated in *OsSRLK*-OX5 than in WT at 0 and 4 DDI, *ossrlk* maintained higher green pigments during DIS compared with those in WT (Figure 3c,d). These chlorophyll contents resulted in relatively lower Chl *a*/*b* ratio in *ossrlk* than in WT at 4 DDI (Figure 3e). These results indicate that *OsSRLK* promotes leaf yellowing by degrading both Chl *a* and Chl *b* during DIS. To identify whether *OsSRLK* affects the expression of genes related to chlorophyll degradation and leaf senescence, we investigated the transcript levels of chlorophyll degradation genes (CDGs) and senescence-associated genes (SAGs) in the detached leaves of WT, *ossrlk*, and *OsSRLK*-OX5 plants during DIS. RT-PCR analysis revealed that no difference was observed in the transcription of CDGs and SAGs among WT, *ossrlk*, and *OsSRLK*-OX5 (Figure S3).

### 2.4. OsSRLK Regulates the Expression of Phytohormone-Related Genes during DIS

**Figure 4:**
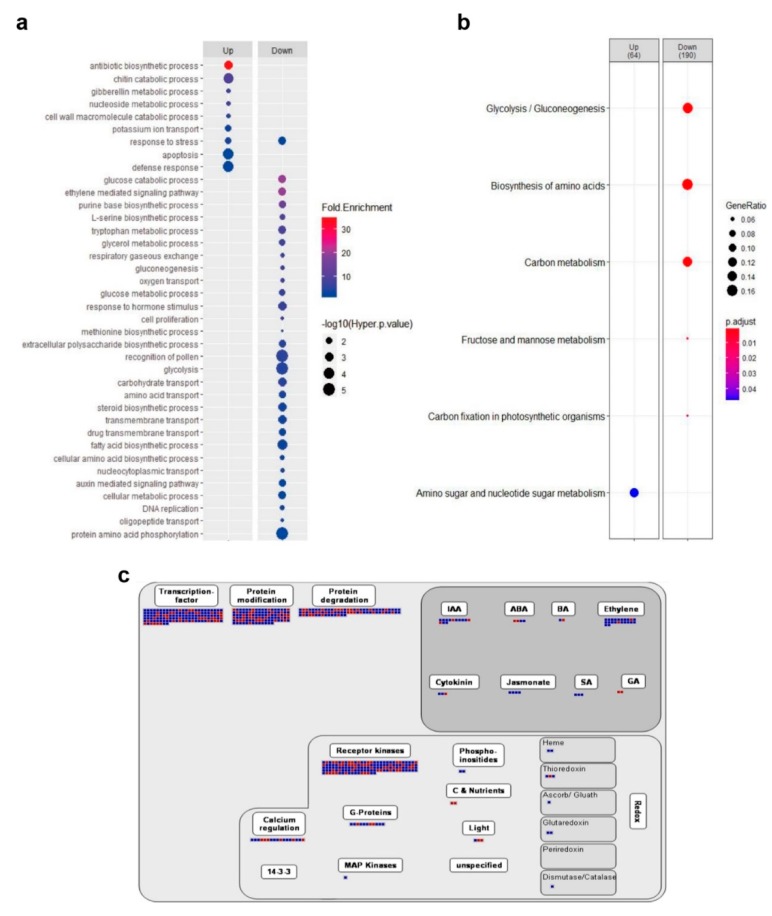
Transcriptome profile of the *ossrlk* mutant. (**a**) Gene ontology (GO) enrichment analysis of the 737 upregulated (Up) and 1303 downregulated (Down) differential expressed genes (DEGs) in *ossrlk* leaves compared to WT during dark-induced senescence (DIS). GO enrichment analysis results were visualized using the ggplot2 package. Gradual change from blue to red dots indicate gradually increased fold enrichment values. Dot size represents statistical significance (−log_10_(hyper *p*-value)). (**b**) Kyoto Encyclopedia of Genes and Genomes (KEGG) enrichment analysis of DEGs. Dot size and color represent the ratio of selected genes to total genes in the pathway and the adjusted *p*-value, respectively. The numbers above the cluster indicate the DEG count for the selected KEGG pathways. (**c**) MapMan analysis of the DEGs. Red and blue squares indicate the members of upregulated and downregulated DEGs, respectively.

To understand the molecular mechanisms of *OsSRLK*-regulated leaf senescence, we profiled genome-wide gene expression in the detached leaves of *ossrlk* and WT plants using RNA sequencing (RNA-seq). Based on the criteria of (log2)-fold-change value >1 and *p*-value <0.05, a total of 2040 differential expressed genes (DEGs), including 737 upregulated genes and 1303 downregulated genes, were selected in *ossrlk* compared to those in WT during DIS. To investigate the function of DEGs, we performed gene ontology (GO) and Kyoto Encyclopedia of Genes and Genomes (KEGG) enrichment analyses. The GO terms in 1303 downregulated DEGs were related to phytohormone signaling pathways, such as “ethylene-mediated signaling pathway”, “response to hormone stimulus”, and “auxin-mediated signaling pathway” (Figure 4a). The KEGG analysis revealed that downregulated DEGs were mainly enriched during the metabolism of glucose, amino acid, carbon, fructose, and mannose (Figure 4b). Furthermore, the MapMan analysis was used to effectively visualize the high-throughput transcriptome data [35]. The results of DEG regulation revealed genes associated with transcription, protein modification and degradation, and phytohormone biosynthesis and signaling (Figure 4c).

Thus, we carried out a literature search on phytohormonal regulation, senescence, and chlorophyll biogenesis to determine the plausible mechanisms of *OsSRLK* action in leaf senescence (Table 1 and Table 2).

For instance, *Oryza sativa* bZIP protein 8 (*OSBZ8*) is induced by ABA treatment and OSBZ8 binds to G-box and G-box-like sequence containing ABA-responsive elements (ABREs) [46]. Stress/ABA-activated protein kinase 6 (SAPK6), a kind of SNF1-related protein kinase 2 (SnRK2), and rice protein phosphatase 2C 51 (OsPP2C51) are involved in the ABA signaling, and their genetic modifications lead to abiotic stress tolerance and seed germination [43,48]. Lipoxygenase (LOX) oxidizes the α-linoleic acid to initiate the JA biosynthesis pathway [80]. Expression of *JAmyb*, a rice R2R3-type MYB transcription factor, is induced by JA treatment [57]. In particular, to identify the *OsSRLK* effect on the phytohormone sensitivity, we evaluated the progress of leaf degreening in the detached leaves of WT, *ossrlk*, and *OsSRL*K-OX5 plants floating on 3 mM MES (pH 5.7) solution containing 5 mM 1-aminocyclopropane-1-carboxylic acid (ACC) or 100 μM methyl jasmonate (MeJA) under continuous light conditions (Figure S4). While leaf greenness was retained for a longer duration in *ossrlk* than in WT at 8 days after treatment (DAT), we did not detect any phenotypic difference between WT and *OsSRLK*-OX5.

In addition to the involvement of *OsSRLK* in phytohormone biosynthesis and signaling, *OsSRLK* regulates the expression of genes that are related to senescence and chlorophyll biogenesis (Table 2). Overexpression of *ONAC106*, a rice NAC transcription factor, delays leaf senescence [76]. The rice mutant harboring nonfunctional *early flowering 8* (*EF8*) allele, encoding a putative HAP3 subunit of the CCAAT-box-binding transcription factor, has more Chl *a* and Chl *b* compared to its parental WT [79]. Collectively, *OsSRLK* is involved in phytohormone biosynthesis and signaling as well as chlorophyll biogenesis. 

### 2.5. Effects of OsSRLK on the Panicle Count Per Plant and Spikelets Per Panicle

**Figure 5:**
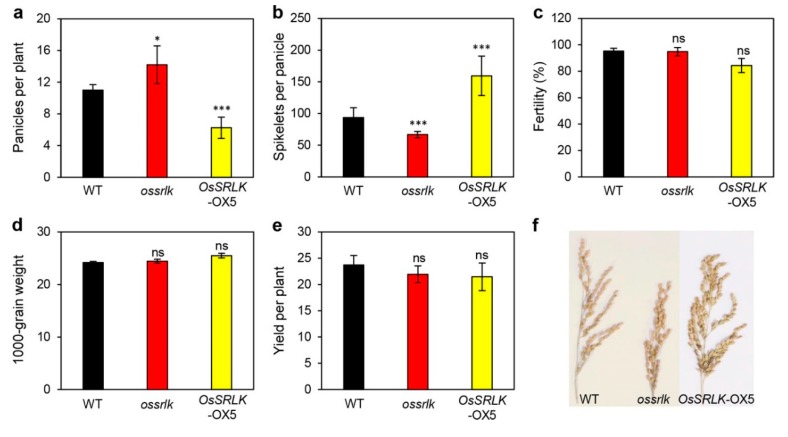
*OsSRLK* affects the panicle count per plant and spikelets per panicle. Agronomic traits of WT, *ossrlk*, and *OsSRLK*-OX5 plants were investigated after harvest in autumn. Comparison of panicles per plant (**a**), spikelets per panicle (**b**), fertility (**c**), 1000-grain weight (**d**), yield per plant (**e**), and phenotype of panicles (**f**) between WT, *ossrlk*, and *OsSRLK*-OX5. Mean and standard deviations were obtained from 10 measurements. Asterisks on *ossrlk* and *OsSRLK*-OX5 indicate a statistically significant difference from WT, as determined by Student’s *t*-test (* *p* < 0.05, *** *p* < 0.001). ns, not significant.

To examine whether *OsSRLK* affects grain production, we evaluated yield components, including panicles per plant, spikelets per panicle, fertility, 1000-grain weight, and grain yield in WT, *ossrlk,* and *OsSRLK*-OX5 grown in the paddy field under NLD conditions. *ossrlk* presented more panicles per plant than WT; however, significantly lesser spikelets were present in each panicle of *ossrlk* compared to that of WT. These interesting traits were in contrast to the overexpression of *OsSRLK*; *OsSRLK*-OX5 revealed less panicles per plant and more spikelets per panicle (Figure 5a,b,f). In contrast to the impact of *OsSRLK* on the panicle and spikelet count, no significant difference was observed in the fertility and 1000-grain weight among WT, *ossrlk*, and *OsSRLK*-OX2 (Figure 5c,d). Thus, regulating the expression of *OsSRLK* did not improve the grain yield due to contradictory effects on the panicle and spikelet count (Figure 5e).

## 3. Discussion

Although the rice genome encodes numerous RLKs that regulate plant development and defense mechanisms, fewer studies are available on RLKs that participate in the regulatory pathways of leaf senescence. In the present study, we found that *OsSRLK*, an LRR-type *RLK* gene, plays a pivotal role in regulating leaf senescence. Expression of *OsSRLK* was induced by both age- and dark-induced leaf senescence (Figure 1b,c), and *ossrlk* mutation by T-DNA insertions retained the leaf greenness during DIS (Figure 2c). These observations suggest the possible roles of *OsSRLK* in chlorophyll degradation. Indeed, more Chl *a* and Chl *b* were present in *ossrlk* than in WT at 3 DDI (Figure 2d). In contrast, overexpression of *OsSRLK* rapidly degraded these green pigments, thereby promoting leaf yellowing compared to WT (Figure 3b,c). Both Chl *a* and Chl *b* are related to the stability of LHCII [81]. Thus, the absence of Chl *b* allows proteases to degrade LHCII [82]. LHCII plays a pivotal role in grana formation [3]. Based on the results obtained from microscopy and immunoblot assay (Figure 2g,h), it could be concluded that, during DIS, high levels of Chl *b* due to *ossrlk* mutation enhance the stability of LHCII and subsequently contribute to intact grana stacks.

Among the senescence regulators affecting chlorophyll degradation, CDGs encode the chlorophyll-degrading enzymes that sequentially catalyze the breakdown of chlorophyll. In the present study, CDG transcript levels in *ossrlk* and *OsSRLK*-OX5 were quite similar to those observed in WT during DIS (Figure S3), indicating that *OsSRLK*-regulated chlorophyll degradation is independent of regulating CDG transcription. Therefore, we explored the alternative regulatory pathways of *OsSRLK*-regulated chlorophyll degradation. Genome-wide analysis using RNA sequencing revealed that phytohormone-related genes were significantly altered in *ossrlk* compared to WT during DIS (Figure 4c). Phytohormones are an important internal factor that determines the onset and progress of leaf senescence [18]. Thus, rice and Arabidopsis plants with genetic modifications in the phytohormone-related genes exhibit delayed or promoted leaf senescence phenotype. For instance, 9-cis-epoxycarotenoid dioxygenase (NCED) is a rate-limiting enzyme involved in ABA biosynthesis, and overexpression of *OsNCED3* accelerates leaf yellowing in rice [83]. Impaired ABA signal transduction by mutation in *ABA insensitive 5* (*ABI5*) inhibits chlorophyll degradation, thereby allowing the mutants to retain leaf greenness [84,85]. In addition, JA is also considered as a senescence inducer. Inaccurate perception of JA in the rice *coronatine insensitive 1* (*oscoi1*) mutant leads to delayed leaf yellowing [86]. Deceased endogenous JA levels due to downregulation of JA biosynthetic genes, such as *LOX* and *allene oxide synthase* (*AOS*), delay leaf senescence in rice [23]. Ethylene is a signaling molecule that promotes leaf senescence. For instance, increased endogenous ethylene can activate ethylene insensitive 2 (EIN2) and EIN3, resulting in precocious leaf senescence [87]. *ossrlk* mutation significantly downregulated the expression of phytohormone-related genes during DIS. These included *OSBZ8*, *SAPK6,* and *OsPP2C51* in ABA signaling; *OsLOX1*, *OsLOX2*, and *JAmyb* in JA biosynthesis and signaling; and *ETR2* and *ETR3* in ethylene signaling (Table 1). Furthermore, the *ossrlk* leaves retained more green pigments than WT under ACC or MeJA treatment (Figure S4). These findings suggest that *OsSRLK* degrades chlorophyll by participating in the phytohormonal regulation of leaf senescence. Plants have developed several RLK signal transduction pathways to regulate phytohormone signaling. For instance, brassinosteroids are perceived by LRRs of BRI1 and form BRI1–BAK1 complex. This allows autophosphorylation on the kinase domains of BRI1, leading to BR signaling [88]. In this scenario, *OsSRLK* may be involved in phytohormone perception as a receptor, and it activates phytohormone signaling that mediates chlorophyll degradation (Figure S5).

Interestingly, mutation of *OsSRLK* increased the panicle count and decreased the spikelet count (Figure 5a,b). Tillering is an important agronomic trait that specifically determines the panicle count per plant in rice [89]. Auxin affects plant architecture, thus determining grain yield components, such as tiller number and panicle morphology [90]. The suppression of rice *PIN-formed 1* (*OsPIN1*) RNA interference increases the tiller count [71]. The downregulation of *OsPIN1* by *ossrlk* mutation may suggest that *OsSRLK* is involved in *OsPIN1*-mediated rice tillering (Table 2).

## 4. Materials and Methods

### 4.1. Plant Materials, Growth Conditions, and Experimental Treatments

The wild-type *Oryza japonica* rice cultivar “Dongjin” (parental line), *ossrlk* and *sgr* mutant plants, and *OsSRLK*-overexpressed transgenic plants were grown in the paddy field under NLD conditions (>14 h sunlight per day) or in a growth chamber under LD conditions (14-h light/10-h dark) in Anseong, Republic of Korea (37° N latitude). The T-DNA insertion mutant *ossrlk* was obtained from Kyung-Hee University, Republic of Korea [91,92]. Rice plants grown in a growth chamber for 3 weeks were used for dark and phytohormone treatments. Detached leaves of rice plants were incubated in 3 mM MES buffer (pH 5.8) with the abaxial side facing upward at 28 °C in complete darkness or in 3 mM MES buffer containing 5 mM ACC or 0.1 mM MeJA under continuous light conditions (40 mmol·m^−2^·s^−1^).

### 4.2. Determination of Chlorophyll Content, Photosynthetic Activity, SPAD Value, and Ion Leakage Rate

Chlorophyll was extracted from the detached leaves of three-week-old plants incubated in complete darkness using 80% ice-cold acetone. Extracts were subjected to centrifugation at 10,000× *g* for 15 min at 10 °C, and the absorbance of the supernatants was then measured at 647 and 663 nm using a UV–VIS spectrophotometer (BioTek instruments, Winooski, VT, USA). Chlorophyll content was calculated as previously described [93,94].

Rice plants grown in a paddy field under NLD conditions were used for determination of photosynthetic activity and SPAD value. The middle portion of the flag leaves was adapted in the dark for 10 min. The Fv/Fm ratio was measured using an OS-30p+ instrument (Opti-Science, Hudson, NH, USA). The SPAD was measured in the flag leaves using a SPAD-502 instrument (Konica Minolta, Tokyo, Japan).

Ion leakage from the detached leaves was measured by subjecting them to dark treatment as described previously with minor modifications [95]. The detached leaves were immersed in 6 mL of 400 mM mannitol for 3 h at 23 °C with gentle shaking, and the initial conductivity was determined with a conductivity meter (CON6 Meter, LaMotte Co., Chestertown, MD, USA). Total conductivity was measured after the samples were incubated at 85 °C for 20 min. The rate of ion leakage was calculated based on the ratio of the percentage of initial conductivity to that of total conductivity.

### 4.3. RNA Isolation and RT-qPCR Analysis

Total RNA was extracted from the leaves using the Total RNA Extraction Kit (Macrogen, Seoul, Korea), according to the manufacturer’s instructions, and RNase-free DNase (iNtRON Biotechnology, Seoul, Korea) was added to eliminate genomic DNA. First-strand cDNA was synthesized from 2 μg of total RNA in a 20 μL volume using Moloney murine leukemia virus (M-MLV) reverse transcriptase (Promega, Madison, WI, USA) and oligo(dT)_15_ primers and was then diluted with 80 μL distilled water. qPCR was performed with gene-specific primers, and the results were normalized to obtain rice ubiquitin 5 (*OsUBQ5*) (Os01g22490) (Table S1) according to the 2^−ΔΔ*C*t^ method [96]. The 20 μL reaction mixture included 2 μL of cDNA, 1 μL of 0.5 μM primers (Table S1), and 10 μL of 2× GoTaq master mix (Promega). The qPCR amplifications were performed using a LightCycler 480 (Roche, Basel, Switzerland) with the following conditions: 94 °C for 2 min followed by 40 cycles at 94 °C for 15 s and 60 °C for 1 min.

### 4.4. Transmission Electron Microscopy Analysis

Transmission electron microscopy was performed according to a previously described method with some modifications [97]. Detached leaves of three-week-old plants were incubated in complete darkness for 0 or 5 days. Small leaf pieces were fixed with modified Karnovsky’s fixative (2% paraformaldehyde, 2% glutaraldehyde, and 50 mM sodium cacodylate buffer, pH 7.2), following which the leaves were washed thrice with 50 mM sodium cacodylate buffer (pH 7.2) at 4 °C for 10 min/wash. The samples were postfixed with 1% osmium tetroxide in 50 mM sodium cacodylate buffer (pH 7.2) for 2 h at 4 °C and washed twice with distilled water at room temperature. Samples were stained en bloc in 0.5% uranyl acetate at 4 °C overnight and then dehydrated in an ethanol gradient solution with propylene oxide, followed by infiltration with Spurr’s resin. Samples were polymerized at 70 °C for 24 h and sectioned with an Ultramicrotome (MT-X, Leica, Wetzlar, Germany). The sections were mounted on copper grids and first stained with 2% uranyl acetate for 7 min and then with Reynolds’ lead citrate for 7 min. Micrographs were obtained using a LIBRA 120 transmission electron microscope (Carl Zeiss, Oberkochen, Germany).

### 4.5. HPLC Analysis

The green pigment was extracted according to the previously described method, and quantitative analyses for Chl *a* and Chl *b* were performed using Prominence UFLC XR (Shimadzu, Kyoto, Japan) with Brownlee SPP C18 column (4.6 × 100 mm, 2.7 um, Perkinelmer, Waltham, MA, USA). The mobile phases were separated using an elution gradient, (A) acetone/methanol (1:4, *v*/*v*) and (B) ion pair reagent/methanol (1:4, *v*/*v*), at a flow rate of 0.6 mL/min. The ion pair reagent was 1 M ammonium acetate in water; the gradient was isocratic A for 4 min, A to B for 5 min, isocratic B for 4 min, and again isocratic A for 2 min. The injected sample (5 μL) was monitored at a wavelength of 650 nm.

### 4.6. RNA-Seq and Analysis

Total RNA for the RNA-seq analysis was extracted from leaves of three-week-old WT and *ossrlk* incubated in complete darkness for 3 days. RNA-Seq was performed with an Illumina HiSeqX instrument (Illumina. San Diego, CA, USA). After trimming the low-quality bases (Q < 20) and short sequence reads (length <20), the trimmed reads were mapped onto a reference rice genome, MSU7 (RGAP, http://rice.plantbiology.msu.edu/, build 7.0), using HISAT2 software (version 2.1.0) [98]. Raw read counts were estimated using featureCounts software (Liao et al. 2013) and normalized with R package, DESeq2 [99] (R version: 3.5.0, DESeq2 version: 1.22.2). The DEGs were estimated with the software package DESeq2, and the genes that exhibited *p*-value < 0.05 and absolute log2 (fold change) ≥1 were determined to be significantly differentially expressed.

We retrieved GO terms related to the biological process from the rice oligonucleotide array database (http://ricephylogenomics-khu.org/ROAD_old/analysis/go_enrichment.shtml, temporary page for updating) [100]. To carry out enrichment analysis, we applied threshold query number of >2, hyper *p*-value < 0.05, and fold enrichment value (query number/query expected number) >2, as previously reported [101,102]. The enriched GO terms were visualized using R studio software and ggplot2 R package (R studio version: 1.1.453, ggplot2 version: 3.0.0). We conducted KEGG enrichment analysis using clusterProfiler package [103] with the organism code “dosa” and a *p*-value cut off of <0.05. Eventually, we visualized the result as a dot plot using the plotting function in the clusterProfiler package and additionally modified the plot using the ggplot2 package. The systemic view of the DEGs was analyzed using MapMan software (v3.6.0 RC1) [104].

### 4.7. Plasmid Construction and Transformation into Rice

To generate the *OsSRLK*-overexpression constructs, full-length *OsSRLK* cDNA was amplified using gene-specific primers listed in Table S1. The amplified *OsSRLK* cDNA was digested with the restriction enzymes *Hind*III and *Hpa*I and inserted into the pGA3426 cloning vector under control of the maize ubiquitin I promoter [105]. The plasmids were transferred into *Agrobacterium tumefaciens* (strain LBA4404) by the freeze–thaw method and were then introduced into calli derived from Dongjin seeds via *A. tumefaciens*-mediated transformation [91].

### 4.8. SDS-PAGE and Immunoblot Analysis

Total proteins were extracted from the detached leaves of three-week-old WT and *ossrlk* incubated in complete darkness. Leaf tissue (10 mg) was homogenized in 100 µL of SDS sample buffer (50 mM Tris, pH 6.8, 2 mM ethylenediaminetetraacetic acid (EDTA), 10% (*w*/*v*) glycerol, 2% sodium dodecyl sulfate (SDS), and 6% 2-mercaptoethanol), and 4 µL of the protein extract was separated by 12% SDS-PAGE (*w*/*v*) and then transferred to an Immobilon-P transfer membrane (Millipore, Burlington, MA, USA). Antibodies against photosynthetic proteins (Lhca1, Lhca2, Lhcb1, Lhcb2, D1, PsaA, PsbC, and PAO) (Agrisera, Vännäs, Sweden) were used for the immunoblot analysis. Horseradish peroxidase activity of secondary antibodies (Sigma, St. Louis, MO, USA) was detected using the ECL system (AbFRONTIER, Seoul, Korea), according to the manufacturer’s instructions.

## 5. Conclusions

We isolated a receptor-like kinase of rice, *OsSRLK*, which is induced by leaf senescence. *OsSRLK* degraded chlorophyll and PSII proteins, thereby promoting leaf yellowing. Mutation of *OsSRLK* led to the downregulation of the genes related to phytohormones, senescence, and chlorophyll biogenesis. Thus, we conclude that *OsSRLK* acts as a positive regulator of senescence.

## Figures and Tables

**Table 1:** Hormone-related genes differentially expressed in *ossrlk* mutant by dark treatment.

Locus_ID (LOC_)	Gene symbol	Fold Change (Log2)	*p*-Value	References
ABA				
Os02g44990	*MAIF1*	2.374286	0.00005	[36]
Os03g57900	*OsiSAP7*	1.427566	0.02315	[37]
Os06g33710	*OsSPDS2*	1.394406	0.01064	[38]
Os11g08210	*OsNAC5*	1.354165	0.03263	[39]
Os02g54160	*OsEREBP1*	−1.019618	0.00204	[40]
Os11g29870	*OsWRKY72*	−1.040532	0.02098	[41]
Os05g49890	*OsRAN2*	−1.061454	0.00769	[42]
Os02g34600	*SAPK6*	−1.221792	0.00969	[43]
Os05g02020	*OsRLCK176*	−1.391996	0.00094	[44]
Os05g41090	*OsCCaMK*	−1.436117	0.00770	[45]
Os01g46970	*OSBZ8*	−1.510641	0.04456	[46]
Os06g51070	*ONAC095*	−1.621916	0.03699	[47]
Os05g49730	*OsPP2C51*	−2.253431	0.02230	[48]
Os03g03370	*DSM2*	−2.439699	0.00472	[49]
Os07g43530	*OsbHLH1*	−2.468823	0.00065	[50]
Os01g01660	*OsIRL*	−4.027866	0.00066	[51]
JA				
Os04g41680	*OsChia4a*	4.944696	0.00223	[52]
Os08g38990	*OsWRKY30*	1.860935	0.02102	[53]
Os03g03660	*OsCDPK1*	−1.027442	0.02197	[54]
Os07g30970	*OsNDPK1*	−1.148937	0.00489	[55]
Os02g02840	*OsRacB*	−1.201440	0.01898	[56]
Os11g45740	*JAmyb*	−1.409207	0.00710	[57]
Os03g08220	*OsLOX2*	−1.575594	0.00335	[58]
Os03g49380	*OsLOX1*	−2.426267	0.01822	[59]
Ethylene				
Os01g12900	*OsRac1*	2.952107	0.01944	[60]
Os01g51430	*OsRTH1*	−1.087656	0.00187	[61]
Os02g57530	*ETR3*	−1.181857	0.03265	[62]
Os04g08740	*ETR2*	−1.220128	0.04523	[63]
Os02g57720	*RWC3*	−2.322009	0.00321	[64]
Os10g28350	*OsARD1*	−3.650923	0.00420	[65]
Os07g47620	*OsUsp1*	−3.842188	0.00163	[66]
Auxin				
Os04g38570	*OsABCB14*	2.802053	0.03772	[67]
Os01g53880	*IAA6*	1.486924	0.01248	[68]
Os04g57610	*OsARF12*	−1.274296	0.03383	[69]
Os01g07500	*FIB*	−1.395635	0.01820	[70]
Os02g50960	*OsPIN1*	−1.538501	0.00160	[71]
Os06g48950	*OsARF19*	−1.720215	0.00083	[72]
Os01g42380	*Ospdr9*	−2.046885	0.00035	[73]
Os07g40290	*OsGH3.8*	−2.265828	0.00652	[74]
Os05g47840	*OsIPT7*	−2.424619	0.04842	[75]

**Table 2:** Senescence-related and chlorophyll biogenesis genes differentially expressed in *ossrlk* mutant by dark treatment.

Locus_ID (LOC_)	Gene Symbol	Fold Change (Log2)	*p*-Value	References
Senescence				
Os08g33670	*ONAC106*	0.611718	0.01580	[76]
Os11g29870	*OsWRKY72*	−1.040532	0.02098	[41]
Os05g02020	*OsRLCK176*	−1.391996	0.00094	[44]
Chlorophyll biogenesis				
Os04g13540	*OsHFP*	4.025662	0.00903	[77]
Os06g14620	*RNRS1*	−1.282421	0.03238	[78]
Os08g07740	*EF8*	−2.647314	0.00042	[79]

## Supplementary Materials

The following are available online at https://www.mdpi.com/1422-0067/21/1/260/s1, Figure S1: Amino acid sequence alignment of RLK protein. Figure S2: The senescence phenotype of *ossrlk* under natural senescence conditions in the field. Figure S3: Expression of CDGs and SAGs in *ossrlk* and *OsSRLK*-OX5 during DIS. Figure S4: Phytohormone hyposensitivity of *ossrlk*. Figure S5: Proposed model for the role of *OsSRLK* in leaf senescence. Table S1: Primers used in this study.

## Abbreviations

WT	wild type
Chl	chlorophyll
NLD	natural long day
LD	long day
RT	reverse transcriptase
DIS	dark-induced senescence
DDI	day(s) of dark incubation
CDGs	chlorophyll degradation genes
SAGs	senescence-associated genes
DEGs	differential expressed genes
