# Supplementary material for: Rice Senescence-Induced Receptor-Like Kinase (OsSRLK) Is Involved in Phytohormone-Mediated Chlorophyll Degradation
Source: Int J Mol Sci. 2019 Dec 30;21(1):260. doi: 10.3390/ijms21010260 (PMC6982081; doi:10.3390/ijms21010260)
Supplement: Supplementary file 1 [file ijms-21-00260-s001.zip › ijms-665208-SI.pdf]

Supplementary

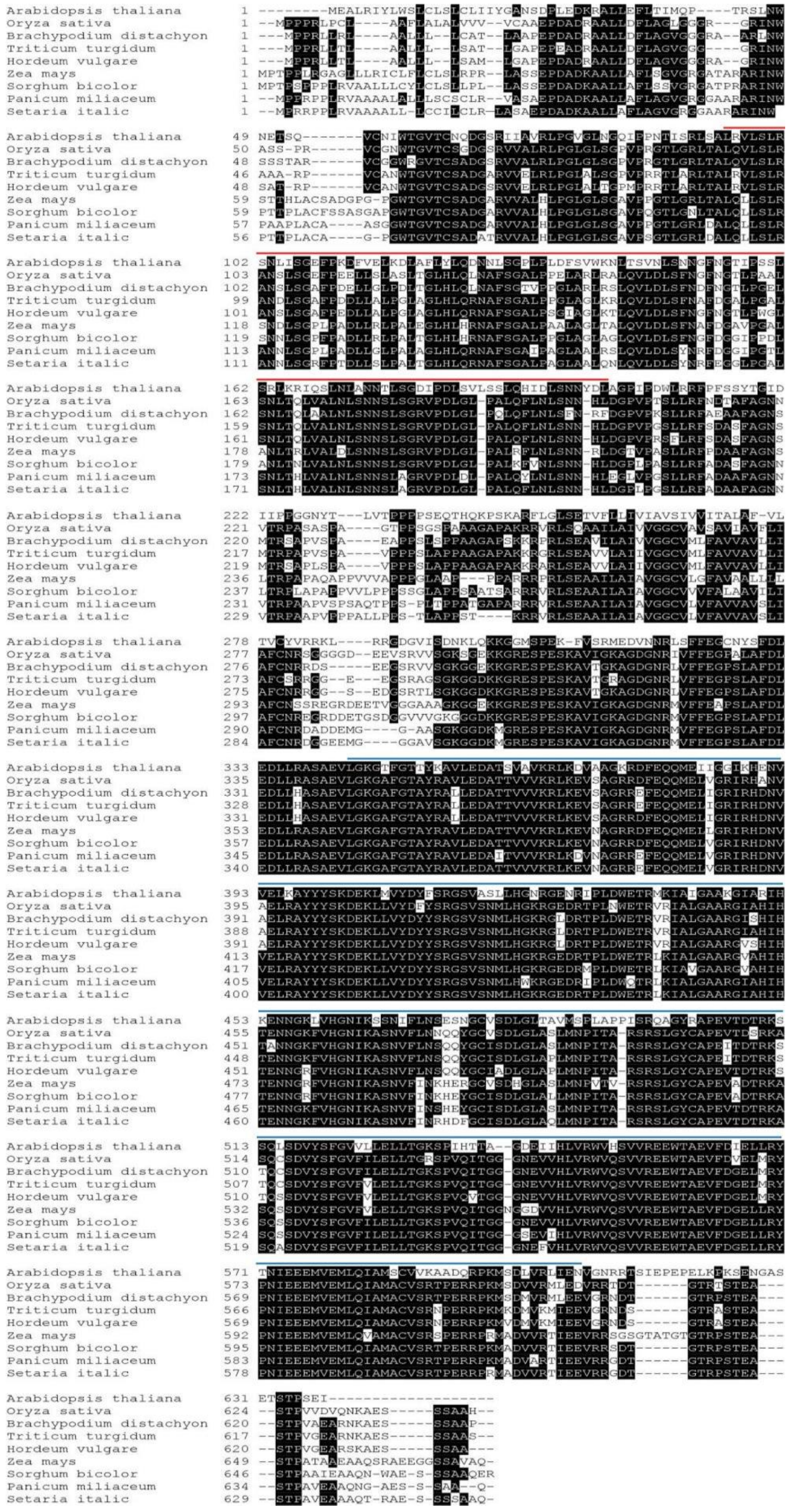

Figure S1. Amino acid sequence alignment of RLK protein. The amino acid sequences for OsSRLK proteins of other plant species homologous to rice SRLK were obtained from NCBI through BLAST analysis. Sequence alignment was performed using ClustalW with default parameters. The sequences are as follows: Arabidopsis thaliana, NM_118505.5; Oryza sativa, LOC_Os01g12390.1; Brachypodium distachyon, XP_003565740; Triticum turgidum, VAH59131; Hordeum vulgare, BAJ86409; Zea mays, NP_001168844; Sorghum bicolor, XP_021312403; Panicum miliaceum, RLN23550; Setaria italic, XP_004967473. Red and blue bars represent the leucine rich repeats and catalytic domain of serine/threonine kinases, respectively.

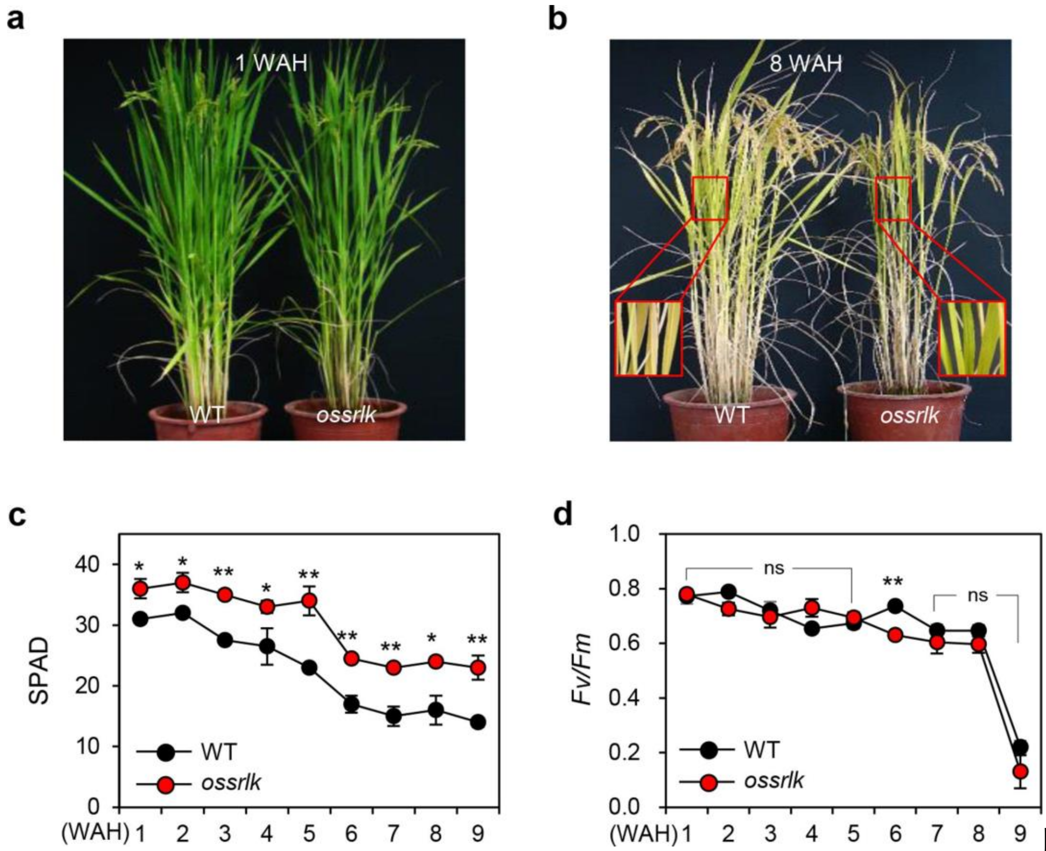

[1]

Figure S2. The senescence phenotype of *ossrlk* under natural senescence conditions in the field. The plants were photographed 1 week after heading (WAH) (a) and 8 WAH (b). SPAD value (c) and *Fv*/*Fm* (efficiency of photosystem II) (d) were measured in WT and *ossrlk* every 1 week from 1 WAH to 9 WAH. Mean and standard deviation values were obtained from 10 measurements. Asterisks indicate a significant difference between WT and *ossrlk* mutant (Student's *t*-test, *$P < 0.05$, **$P < 0.01$). n.s., not significant.

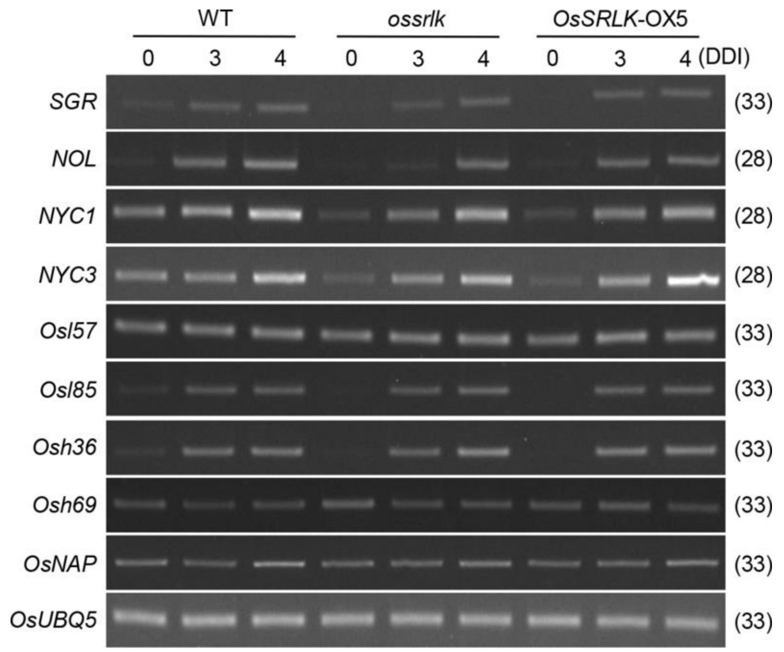

Figure S3. Expression of CDGs and SAGs in *ossrlk* and *OsSRLK*-OX5 during DIS. Total RNA was isolated from the detached leaves of WT, *ossrlk*, and *OsSRLK*-OX5 plants under DIS, as presented in Figure 2 (2 c,h). Transcripts of CDGs (*SGR*, *NOL*, *NYC1*, and *NYC3*) and SAGs (*Osl57*, *Osl85*, *Osh36*, *Osh69*, and *OsNAP*) were determined by reverse-transcriptase PCR analysis. *OsUBQ5* was used as a loading control. Numbers in parentheses indicate the numbers of PCR cycles. DDI, day(s) of dark incubation.

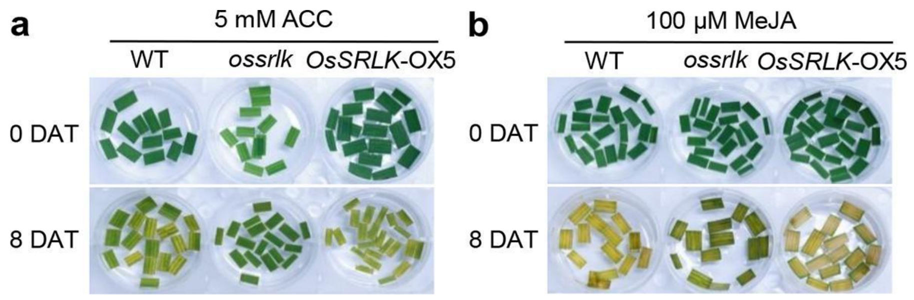

Figure S4. Phytohormone hyposensitivity of *ossrlk*. Detached leaves of 3-week-old WT, *ossrlk*, and *OsSRLK*-OX5 plants were treated with 3 mM MES buffer (pH 5.8) containing 5 mM ACC (1-aminocyclopropane-1-carboxylic acid) (a) and 100 μM MeJA (b) under continuous light conditions at 28 °C. DAT, day(s) after treatment.

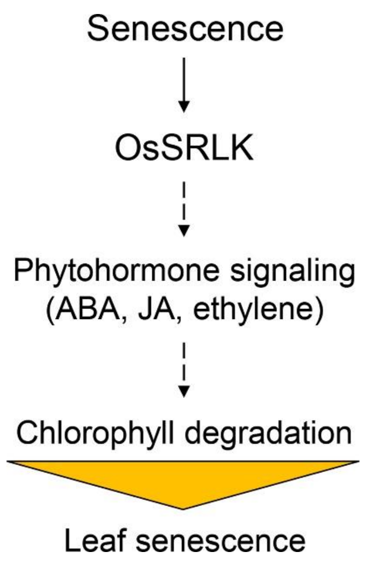

Figure S5. Proposed model for the role of OsSRLK in leaf senescence. Solid and dashed arrows represent direct and indirect activation, respectively.

Supplemental Table S1. Primers used in this study

A. Primers for verification of T-DNA insertion

Primer name	Left primers (5`→ 3`)	Right primers (5`→ 3`)
PFG_1A-15835	TCTCCCTCATCAAAACGTCC	AGTGAGAGGAGCTCCTCCG
pGA2717	ACGCTGAACTTGTGGCCGTT	AACGCTGATCAATTCCACAG

B. Primers for RT-qPCR

Genes	Forward primers (5`→ 3`)	Reverse primers (5`→ 3`)
OsSRLK	GACGTCGAGCTGATGAGGTA	CACCACGTCCGACATCTTAG
OsUBQ5	ACCACTTCGACCGCCACTACT	ACGCCTAAGCCTGCTGGTT

C. Primers for RT-PCR

Genes	Forward primers (5`→ 3`)	Reverse primers (5`→ 3`)
OsSRLK	TTGTTGGACAGATTCAAGAA	ATAGAGACTACTGTGAAACA
SGR	AGGGGTGGTACAACAAGCTG	GCTCCTTGCGGAAGATGTAG
NOL	GAAAGGGTAGAATCTGCGGTG	CTGCAGAGATTTTGTAAGGTG
NYC1	GAATCCGTAATTGGGCTGAA	CTGGAAGAGGTCCACCTGAG
NYC3	TGCTGCATCCTGTCCACACCTTG	GATGCAAATGATGCAGCAGCTGC
Osl57	ACCCTAAAGTAAATGAAGTC	CCTGCTCTTGTCTTGTTA
Osl85	CGTCACGGACACGTTCGC	GCAAGAACATGGCTGCTGC
Osh36	GTTGAGGCGATGGTCAACC	CAGTGTAAAGCCGGGCAATC
Osh69	CCTGCTCTTGTCTTGTTA	GGTGAACACTATGGAACA
OsNAP	AACCATTTCATCGCGAACAAC	CAGTGACGATCCCTGCAAGG
OsUBQ5	ACCACTTCGACCGCCACTACT	ACGCCTAAGCCTGCTGGTT

D. Primers for plant transformation

Primer name	Forward primer (5`→ 3`)[a]	Reverse primer (5`→ 3`)[a]
OsSRLK_*HindIII* and *HpaI*	AAGCTTATGCCGCCGCCGAGGCTG	GTTAACTCAGAGGAGCTCCTCCG

[a]The underlined nucleotides represent the restriction site for restriction enzymes.
